# Impact on short-lived climate forcers increases projected warming due to deforestation

**DOI:** 10.1038/s41467-017-02412-4

**Published:** 2018-01-11

**Authors:** C. E. Scott, S. A. Monks, D. V. Spracklen, S. R. Arnold, P. M. Forster, A. Rap, M. Äijälä, P. Artaxo, K. S. Carslaw, M. P. Chipperfield, M. Ehn, S. Gilardoni, L. Heikkinen, M. Kulmala, T. Petäjä, C. L. S. Reddington, L. V. Rizzo, E. Swietlicki, E. Vignati, C. Wilson

**Affiliations:** 1 Institute for Climate and Atmospheric Science, School of Earth and Environment, University of Leeds, Leeds, UK; 2 Cooperative Institute for Research in Environmental Sciences, University of Colorado, Boulder, CO USA; 3 Chemical Sciences Division, NOAA Earth System Research Laboratory, Boulder, CO USA; 4 Department of Physics, University of Helsinki, P.O. Box 64, 00014 Helsinki, Finland; 5 Institute of Physics, University of Sao Paulo, Diadema, Sao Paulo Brazil; 6 National Research Council, Institute of Atmospheric Sciences and Climate, Bologna, Italy; 7 Department of Earth and Exact Sciences, Institute of Environmental, Chemical and Pharmaceutics Sciences, Federal University of Sao Paulo, UNIFESP, Diadema, Sao Paulo Brazil; 8 Division of Nuclear Physics, Lund University, P.O. Box 118, 221 00 Lund, Sweden; 9 Centre for Environmental and Climate Research, Lund University, P.O. Box 118, 221 00 Lund, Sweden; 10 European Commission, Joint Research Center, Directorate for Energy Transport and Climate, Ispra, Italy

## Abstract

The climate impact of deforestation depends on the relative strength of several biogeochemical and biogeophysical effects. In addition to affecting the exchange of carbon dioxide (CO_2_) and moisture with the atmosphere and surface albedo, vegetation emits biogenic volatile organic compounds (BVOCs) that alter the formation of short-lived climate forcers (SLCFs), which include aerosol, ozone and methane. Here we show that a scenario of complete global deforestation results in a net positive radiative forcing (RF; 0.12 W m^−2^) from SLCFs, with the negative RF from decreases in ozone and methane concentrations partially offsetting the positive aerosol RF. Combining RFs due to CO_2_, surface albedo and SLCFs suggests that global deforestation could cause 0.8 K warming after 100 years, with SLCFs contributing 8% of the effect. However, deforestation as projected by the RCP8.5 scenario leads to zero net RF from SLCF, primarily due to nonlinearities in the aerosol indirect effect.

## Abstract (web summary)

The climate impacts of deforestation due to changes in biogenic volatile organic compound emissions, which act as short-lived climate forcers (SLCFs), are poorly understood. Here the authors show that including the impact SLCFs increases the projected warming associated with idealised deforestation scenarios.

## Introduction

Forests cover almost one third of the Earth’s land area and their distribution is changing as a result of land-use change (LUC). Since the Industrial Revolution the increased demand for agricultural land has resulted in the conversion of forests to crops and pastures over large parts of the world^1^. Rapid deforestation continues today with mean annual global forest loss of 0.19 million km^2^ between 2000 and 2012 driven by continued and intensifying deforestation in the tropics^2^. In the Amazon, 20% of the original forest has now been cleared^3^ and in Borneo forest cover declined by 31% between 1973 and 2010^4^. In other parts of the world, natural forest regrowth due to agricultural abandonment, as well as afforestation, has led to substantial regional increases in forest cover^2^. Future land cover trajectories may exacerbate or help to mitigate climate change. A recent assessment suggests that avoided deforestation, forest restoration and other land management options, could provide 37% of the cost effective carbon dioxide (CO_2_) mitigation needed before 2030 to limit future warming to 2 K above pre-industrial levels^5^. The formulation of sound climate mitigation policy^6^ for the next century requires a robust understanding of the climate impacts associated with the current wide range of future LUC projections^7^.

Numerous biogeophysical and biogeochemical interactions between vegetation and the atmosphere^8–11^ complicate the climate impacts of LUC. Deforestation often results in the direct emission of CO_2_ through forest burning or decay of wood. This emission acts to increase the concentration of CO_2_ in the atmosphere^12^, resulting in a positive radiative effect, or warming, on climate. Forests are darker in colour than grass or cropland so deforestation increases the reflectivity of the land surface (i.e., its albedo). An increase in surface albedo will exert a negative radiative effect, or cooling, on the climate^8^. The net impact of these biogeochemical and biogeophysical effects depends strongly upon forest latitude: tropical deforestation is generally found to warm the climate whereas high latitude deforestation is generally found to cool the climate^8–10,13^.

In addition to these effects, forests and vegetation emit biogenic volatile organic compounds (BVOCs) into the atmosphere. Rates of BVOC emission will be affected by LUC; this can affect climate by changing the concentrations of short-lived climate forcers (SLCFs) including aerosols, ozone (O_3_) and methane (CH_4_). Once emitted, BVOCs are rapidly oxidised by O_3_, the hydroxyl radical (OH) and the nitrate radical (NO_3_), affecting the oxidative capacity of the atmosphere. Any change to the oxidative capacity of the troposphere will affect the concentration of two important greenhouse gases, O_3_ and CH_4_. In the presence of nitrogen oxides (NO_x_), VOCs also contribute to O_3_ production in the troposphere, complicating their impact on climate^14^.

Low-volatility products from BVOC oxidation can participate in new particle formation^15,16^ as well as condensing onto existing particles in the atmosphere and aiding their growth to larger sizes^17,18^. Through these processes, secondary organic aerosol (SOA) formed by VOC oxidation, influences the number of climatically relevant particles in the atmosphere. Once particles have grown to a dry diameter of approximately 100 nm they can interact directly with incoming shortwave radiation (exerting a direct radiative forcing or effect) and act as condensation nuclei for the formation of cloud droplets (resulting in an indirect radiative forcing or effect). Through these direct and indirect radiative effects, the presence of biogenic SOA likely exerts a negative radiative effect on the climate^19,20^.

Most assessments of the climate impacts of LUC have been restricted to CO_2_ and biophysical impacts^8–10,13^. Only recently^21–24^, have assessments of the overall climate impact of LUC considered the impacts on SLCFs. A study^22^ of the impacts of historical LUC on SLCFs, suggested that reductions in BVOCs due to LUC have reduced O_3_ and CH_4_ concentrations resulting in a climate cooling; however, this study did not fully evaluate aerosol radiative effects. Whilst other studies have assessed the impacts of complex historical^22–25^ or future LUC^24,26^, our approach is to conduct idealised deforestation scenarios, allowing us to isolate the impact of this specific LUC on climate. Our focus is on the impact of deforestation on SLCFs via changes in BVOC emissions, so we do not include trace gas emissions associated with the agricultural activities that may occur on deforested land.

To further explore the implications of LUC on the production of SLCFs, we conducted idealised experiments of global, boreal (90–50°N), temperate (50–20°N and 20–50°S) and tropical (20°N–20°S) deforestation, consistent with previous studies of the CO_2_ and biogeophysical impacts of LUC^9^. To estimate the radiative impacts of deforestation we combine a land-surface model with a chemical transport model, global aerosol model, and radiative transfer model (see Methods). We also consider a level of future deforestation consistent with the RCP8.5 scenario. We focus on changes to forest cover in the present-day and future, since this is most relevant for climate change mitigation^5^.

We quantify the radiative forcing (RF) due to deforestation through changes in the concentration of SLCFs and compare these to the CO_2_ and biogeophysical impacts that are more routinely calculated. It is found that SLCFs contribute a net positive RF and enhance the warming associated with idealised large-scale deforestation.

## Results

### Evaluation against atmospheric observations

We evaluated simulated aerosol at boreal and tropical forest locations which are strongly influenced by the emission of BVOCs and less strongly perturbed by anthropogenic pollution^27^ (Supplementary Discussion). In boreal forest regions, the model underestimates both the number of cloud droplet forming particles and aerosol mass concentrations, with a contrasting overestimate of both parameters in tropical forest regions (Supplementary Fig. 1). To explore this issue we conducted an additional simulation where we increased the production of SOA from BVOCs by a factor of 5 in boreal latitudes and decreased the production of SOA by a factor of 2 at tropical latitudes, resulting in better agreement with observations at both locations. Applying these modified SOA yields reduces the simulated global SOA burden from 40 Tg SOA a^−1^ to 31 Tg SOA a^−1^ (Supplementary Table 1), still within the large range of uncertainty associated with the global source of biogenic SOA^28^. Uncertainty in aerosol formation from BVOCs typically contributes less than 10% of total uncertainty in the simulated number of cloud droplet forming particles in our model^29^, making it difficult to attribute model bias specifically to issues associated with SOA formation. However, we find limited sensitivity of aerosol RF to changes in SOA burden particularly for the aerosol indirect effect (Supplementary Table 2); we therefore report results from our deforestation experiments with standard SOA yields.

### Simulated impacts of deforestation

Simulated global deforestation reduces isoprene emission by 87% and monoterpene emission by 94% (Supplementary Table 1), with the remaining emission coming from shrubs, crops and the grasses that replaced the forests. Most of this reduction in emission is due to tropical deforestation, which reduces isoprene and monoterpene emissions by 72 and 74% respectively. The reduction in BVOC emission due to simulated global deforestation reduces SOA production by 91% (Supplementary Table 1), with tropical deforestation accounting for 80% of the global reduction in SOA formation.

**Fig. 1:**
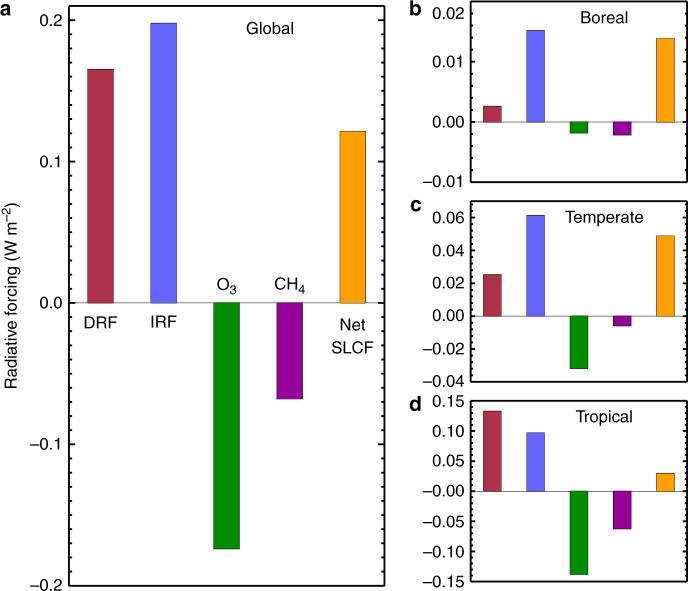
Global annual mean radiative forcings due to changes in the concentrations of short-lived climate forcers (SLCFs) under global (**a**) and regional (**b**–**d**) deforestation scenarios. Bars represent the net radiative forcing from SLCFs (orange) and the aerosol direct radiative forcing (DRF; in red), first aerosol indirect radiative forcing (IRF; in blue) and RF due to changes in O_3_ (green) and CH_4_ (purple)

**Fig. 2:**
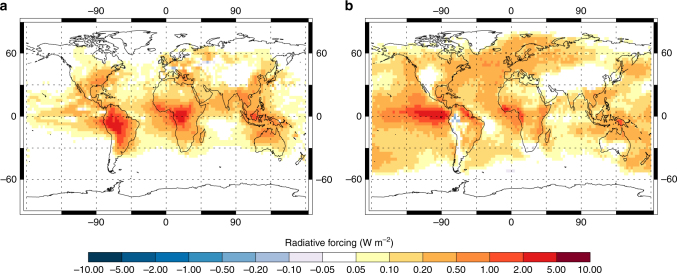
Aerosol radiative effects due to global deforestation. Direct radiative forcing (**a**) and first indirect radiative forcing (**b**)

Figure 1 summarises the annual mean RFs due to changes in the concentrations of SLCFs under global and regional deforestation scenarios. Global deforestation results in a positive global annual mean aerosol direct radiative forcing (DRF) of 0.17 W m^−2^ (all values also given in Supplementary Table 2). This positive RF occurs due to the reduced production of biogenic SOA, meaning that fewer particles grow large enough to interact directly with radiation in the atmosphere. The largest DRF occurs over tropical regions, exceeding 2 W m^−2^ over the Amazon and Congo forests (Fig. 2 and Supplementary Fig. 2). The different scenarios highlight a latitudinal sensitivity to deforestation, with tropical deforestation accounting for 80% of the DRF due to global deforestation.

Global deforestation causes a global annual mean first aerosol indirect radiative forcing (IRF) of 0.20 W m^−2^, due to a reduction in the global annual mean concentration of particles able to form cloud droplets. The spatial pattern of the IRF is more complex than the DRF (Fig. 2) but the strongest IRF also occurs at tropical latitudes due to substantial year-round decreases in cloud droplet forming particles (up to 50%) and the abundance of low-level clouds over the tropical coasts and adjacent ocean regions.

The strongest first IRF per change in SOA produced (10 mW m^−2^ Tg(SOA)^−1^) comes from temperate (20–50°N and 20–50°S) deforestation, which reduces global SOA production by only 15%, but leads to a strong positive RF over remote ocean regions with high cloud cover. Boreal deforestation reduces the global annual mean concentration of particles able to form cloud droplets by only 1.4%, but regional reductions over northern Russia and Canada in the NH summertime exceed 30%. The combined contribution of temperate and boreal deforestation leads to a Northern hemisphere summertime (June-July-August mean) first IRF of more than 0.3 W m^−2^ across much of the region between 40 and 80°N (Supplementary Fig. 3).

In the present-day atmosphere, NO_x_ concentrations are sufficiently high in most locations that BVOCs, particularly isoprene, contribute to the production of O_3_. As such, the reduction in BVOC emissions associated with global deforestation leads to a decrease in surface O_3_ concentration across much of the planet (Supplementary Fig. 4). However, in more pristine locations, such as over the Amazon rainforest, modelled NO_x_ concentrations are low and direct reaction of BVOCs with O_3_ out-competes O_3_ production from BVOC oxidation. In these locations, the reduction in BVOC emission associated with deforestation leads to an increase in annual mean O_3_ concentrations at the surface (up to 11 ppbv; Supplementary Fig. 4).These increases diminish with altitude and the zonal mean change in O_3_ throughout the troposphere is negative at all latitudes (Supplementary Fig. 5).

Global deforestation leads to a global annual mean tropospheric O_3_ radiative forcing of −0.17 W m^−2^. The strongest O_3_ RF is simulated for tropical deforestation (−0.14 W m^−2^). This is due to the relatively large perturbation to global BVOC emissions in a region where efficient convective transport of air masses from the surface to the upper troposphere enhances the impact of changes to O_3_ precursors, and because subsequent changes in O_3_ occur in the radiatively important tropical tropopause region^30^. Our simulated O_3_ RF due to global deforestation, is lower in magnitude than previous estimates^31^ of the radiative effect due to BVOC emission in the present day (0.5 W m^−2^); this could arise from differing model sensitivities to perturbations in O_3_ precursors, or the radiative effect per O_3_ change.

The reduction in BVOC emission associated with global deforestation leads to an increase in annual tropospheric mean OH concentration, from 1.36 × 10^6^ to 1.46 × 10^6^ molecules cm^−3^, which reduces the lifetime of CH_4_ from 7.6 years to 7.1 years. This change in CH_4_ lifetime is used to diagnose a reduction in steady-state CH_4_ concentration of 180 ppb due to global deforestation, and a RF of −0.07 W m^−2^. However, it is important to note that uncertainties in the consumption of OH during isoprene oxidation^32,33^ will influence the sensitivity of CH_4_ to changing BVOC emissions.

We calculate the combined impact of deforestation on the concentration of SLCFs through the combination of aerosol (DRF and first IRF), O_3_ and CH_4_ RFs. The combined RF from SLCFs is a balance between a warming aerosol RF and a cooling due to reductions in O_3_ and CH_4_. We estimate that global deforestation causes an overall positive RF of 0.12 W m^−2^ due to changes in SLCFs (Fig. 1). Our study demonstrates the importance of accounting for aerosol-cloud effects; when we ignore the first IRF, our combined SLCF RF is negative (−0.08 W m^−2^). Previous studies of the impact of deforestation or LUC on SLCFs that did not include the first IRF (e.g. ref.^22)^, may therefore have attributed too much of a negative RF, or cooling effect, to changes in SLCFs from deforestation.

Figure 1 also shows the global RF due to regional deforestation simulations. The combined global mean SLCF RF due to tropical (0.03 W m^−2^), temperate (0.05 W m^−2^) and boreal (0.01 W m^−2^) deforestation are positive, due to the strong positive aerosol RFs in comparison to weaker negative O_3_ and CH_4_ RFs. Previous work, that accounted only for biogeophysical changes, has shown that the impact of temperate deforestation is seasonally dependent, causing a local summertime warming and a wintertime cooling^13,34^. Our analysis suggests that the effect of SLCFs would enhance this summertime warming. The impact of these temporally and spatially inhomogeneous RFs on regional climate may be important and needs to be explored in future work.

We also assessed the impact of deforestation that is consistent with the RCP8.5 scenario. Under this scenario the combined global mean SLCF RF is 0.00 W m^−2^ due to a warming aerosol RF being offset by cooling from changes in O_3_ and CH_4_. The DRF, O_3_ and CH_4_ RF have a relatively linear response to changes in SOA, with SOA reductions of 4% in the RCP8.5 scenario vs 91% under global deforestation. In contrast, the IRF efficiency is reduced from +5.4 mW m^−2^ Tg(SOA)^−1^ under global deforestation to −0.5 mW m^−2^ Tg(SOA)^−1^ under the RCP8.5 scenario. This further highlights the non-linear responses between changes in SOA and IRF that were calculated in the regional deforestation experiments.

We calculate the natural variability of SLCF RF using an 11-year GLOMAP simulation for the period 1997 to 2007. We calculate the standard deviation in the annual mean RE as 0.025 W m^−2^ for DRF and 0.017 W m^−2^ for IRF. This means that radiative impacts from global and regional deforestation (Supplementary Table 2) typically exceed natural variability, whereas the impact from the RCP8.5 scenario is substantially less than natural variability.

To assess the relative importance of the calculated RFs of changes to SLCFs from LUC they need to be compared to the RFs due to surface albedo change and CO_2_ emission (Supplementary Fig. 6; CO_2_ concentration values taken from ref.^9^), summarised in Table 1. We assume permanent land-use changes such that the RFs associated with changes to SLCFs and surface albedo are constant with time (i.e., from a change occurring in year 1 and persisting). The CO_2_ concentration, and associated RF, evolves over time as the carbon pools change (see Methods). We note that taking the CO_2_ concentration pathways from ref.^9^ may introduce slight differences in the distribution of plant functional types across the land-surface, from that used to calculate the SLCF and albedo RFs.

**Table 1:** Summary of global annual mean radiative forcings due to idealised deforestation scenarios

	RF due to Δ[CO_2_]^a^ (W m^−2^)	RF due to Δalbedo (W m^−2^)	RF due to Δ SLCFs (W m^-2^)	Combined RF due to CO_2_ (after 100 years), albedo and SLCFs (W m^-2^)
Boreal deforestation	0.01	−0.51	0.01	−0.49
Temperate deforestation	0.71	−0.27	0.05	0.49
Tropical deforestation	1.26	−0.18	0.03	1.11
Global deforestation	2.22	−0.96	0.12	1.38
RCP8.5 level deforestation in 2100	0.24^b^	0.02^b^	0.00	0.26

Global annual mean radiative forcings (W m^−2^) due to change to CO_2_ concentration, albedo and SLCFs, and the net RF due to CO_2_, albedo and SLCF changes, for each deforestation scenario

^a^For idealised deforestation scenarios, CO_2_ RF is diagnosed 100 years after the initial deforestation

^b^RF for CO_2_ and albedo change due to land-use change under RCP 8.5 taken from ref.^24^

The global mean RF due to surface albedo change (−0.96 W m^−2^) under global deforestation is dominated by the impact of boreal deforestation, which alone exerts a global annual mean RF of −0.51 W m^−2^. Consistent with previous studies^8,24^, the increase in surface albedo, and enhanced snow cover, due to boreal deforestation leads to strong regional RFs, up to −25 W m^−2^ (not shown). Despite the large area affected, tropical deforestation results in a much smaller albedo change and a global annual mean RF of −0.18 W m^−2^.

Simulated global deforestation in 2000 leads to a rapid increase in CO_2_ RF that peaks in 2080 and gradually declines afterwards (Supplementary Fig. 7)^9^. The global mean atmospheric CO_2_ concentration remains approximately 380 ppm above the baseline after 100 years, giving a RF of 2.22 W m^−2^ (Table 1). The CO_2_ changes come nearly entirely from deforestation at tropical and temperate latitudes; note that the carbon cycle model response is not completely linear so global deforestation has a larger effect than the sum of the regional components.

We estimate a combined RF due to global deforestation of 1.38 W m^−2^ (100 years after deforestation, Table 1); including SLCFs increases the positive RF by 10%. This enhancement from SLCFs is less than a previous study that also included CH_4_ and N_2_O emissions from agricultural activities after the initial LUC^24^. Boreal deforestation has a negative combined RF due to strong surface albedo effects, whereas temperate and tropical deforestation have a positive combined RF.

### Temperature change due to deforestation

**Fig. 3:**
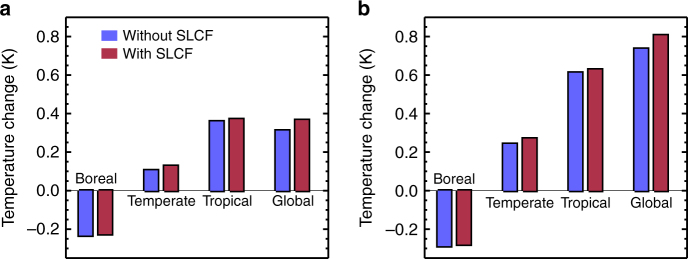
Temperature changes due to deforestation. Net temperature changes after 20 years (**a**) and 100 years (**b**) for the global and regional deforestation scenarios, with and without the impacts of SLCFs

Standard metrics used to compare RFs are Global Warming Potentials (GWP) based on time integrated RFs, or Global Temperature Potential (GTP) based on end point global surface temperature change estimates^35^. Here we chose to compare GTP after 20 and 100 years, assuming a mid-range equilibrium climate sensitivity. Our calculation of GTP ignores regional climatic implications, which may be particularly important for LUC^36^ and SLCFs where RFs are spatially inhomogeneous. The use of a single climate sensitivity to translate radiative forcing into global temperature change ignores the possibility that spatially variable forcings might have a different response to that of carbon dioxide and does not account for differences in the rapid adjustments across mechanisms^37^. Figure 3 shows the net impact of global, boreal, temperate and tropical deforestation, highlighting the contribution of SLCFs. Global deforestation leads to 0.36 K global surface warming after 20 years and 0.8 K warming after 100 years, when the effects of SLCFs are included.

For the case of global deforestation, including SLCFs increases the warming impact of deforestation (compared to that from CO_2_ and surface albedo alone) by around 0.05 K, providing 14% of the total warming after 20 years, and 8% of the total warming after 100 years. As found previously^8–10^, boreal deforestation exerts a global net cooling (approximately 0.3 K after 100 years) due primarily to the dominant effect of increased surface albedo. Including the impacts in SLCFs reduces the magnitude of this net negative RF (from changes to CO_2_ and surface albedo alone) by approximately 3%. We find that tropical deforestation results in a strong net global mean warming effect, consistent with previous studies^9^; including the impacts of SLCFs has a small effect on top of this.

## Discussion

Our analysis confirms the need to differentiate between tropical and boreal LUC when considering climate impacts; even though our scenarios remove forests completely, they are suggestive that future deforestation outside of high latitudes is likely to result in a warming.

Extensive land-use change will dramatically alter fire activity with associated changes in emissions of trace gases and aerosol and climate^22,26^. Here we assumed that fire emissions continue following the initial deforestation, but future work needs to explore a representation of the complex relationships between deforestation and fire.

Further work is required to fully constrain the impacts of BVOCs on SLCFs. BVOC emission fluxes^38^ are uncertain, particularly in the tropics and for reactive BVOCs such as sesquiterpenes^39^. The tropospheric oxidation of BVOCs^32,33^ and their role in SOA formation^40^ requires further elucidation, particularly in low NO_x_ environments. Uncertainties also remain in our understanding of new particle formation mechanisms^16^, the interaction of biogenic oxidation products with other atmospheric constituents^41^ and aerosol microphysical processes^18^. Our understanding of the impacts of deforestation on CO_2_ concentrations and surface albedo are also subject to uncertainty, with estimates of the climate impact from both effects varying by up to a factor of two^24,35^. Future work is needed to quantitatively evaluate the uncertainty across the full range of biogeochemical and biophysical effects. Nevertheless, our study suggests that tropical deforestation would still warm the climate even if we assume a much larger role for SLCFs, such as the strongly negative O_3_ RF that was calculated in another study^22^.

Land-use change has substantial impacts on climate through biogeophysical and biogeochemical effects. By combining the RFs due to CO_2_, surface albedo and SLCFs we find that global deforestation could lead to around 0.8 K warming after 100 years, assuming a mid-range climate sensitivity. Tropical deforestation contributes 0.6 K of the warming, whereas boreal deforestation cools the climate by around 0.3 K in our simple scenarios. Policies aimed at reducing tropical deforestation^42^ are therefore likely to reduce future climate warming. Whilst we have isolated the different impact of boreal, temperate and tropical deforestation on climate, policy discussions may need additional information on the sensitivity of the RF and climate impact to the specific location of deforestation. Land-use change may lead to important regional temperature changes^43^. Future work needs to move beyond the radiative forcing and global mean temperature changes reported here. We estimate that including the impacts of deforestation on SLCFs increases the warming from global deforestation by around 0.05 K (comprising 14% of the total warming after 20 years, 8% after 100 years).

## Methods

### Experimental design

To assess the magnitude of the radiative impacts associated with deforestation, we combine a land surface model, used to estimate the impact of deforestation on BVOC emissions, with a global chemical transport model and detailed aerosol microphysics model to calculate the impacts of altered BVOC emissions on the composition of the atmosphere. We then use a radiative transfer model to calculate the radiative impacts of changes to gas-phase and aerosol species, as well as alterations to surface albedo. We carry out idealised simulations of global and regional deforestation to match previous studies, estimating the CO_2_ and biophysical impacts of LUC^9^. In regional deforestation simulations, we separately remove boreal (90–50°N), temperate (50–20°N and 20°S–50°N), and tropical (20°N–20°S) forests. Additionally, we evaluate the impact on SLCFs, through changes in BVOC emissions, of a level of deforestation consistent with the RCP8.5 scenario.

### Land surface model

To quantify the effect of removing forests on the emission of BVOCs, we use the Community Land Model (CLMv4.0; ref.^44)^, which contains the Model of Emissions of Gases and Aerosols from Nature (MEGANv2.1; ref.^38)^.

Here we use the offline configuration of the CLM at a horizontal resolution of 2.5°×1.9°. Precipitation, temperature, solar radiation, wind, atmospheric pressure and specific humidity fields are taken from an observationally derived data set based on NCEP/NCAR re-analysis^45^. All simulations are performed for the year 2000; since the simulations were run without an interactive carbon-nitrogen cycle, a 40 year spin-up period was sufficient to allow the soil moisture of the CLM to establish equilibrium with the driving meteorology.

Vegetation in the CLM is represented by 15 different plant functional types (PFTs), with additional non-vegetated surface in each grid cell. The distribution of PFTs in the CLM is based on MODIS data^46,47^. We simulate a global total monoterpene emission of 140 Tg(C) a^−1^ and an isoprene emission of 480 Tg(C) a^−1^ in our control simulation, consistent with previous emission estimates^38^. In the deforestation scenarios, forested regions of the land-surface were replaced with climatically appropriate grasses. To avoid scaling up potentially inaccurate leaf area indices (LAI) derived from satellite observations of a small initial area of PFT, the LAI for the grass PFTs (used to replace trees) were updated with the relevant latitudinal averages from the CLM land surface data set.

To evaluate the potential impact of future deforestation, we use the level of deforestation occurring between 2005 and 2100, under the RCP8.5 scenario^48^, to scale our present-day BVOC emissions in proportion to the level of forest loss occurring by 2100. This approach isolates the impact of land-use change from other factors that would affect BVOC emissions in 2100, such as changes in temperature and carbon dioxide concentration. To remain consistent with our complete deforestation scenarios, deforested regions in 2100 are effectively replaced by grass in our RCP8.5 scenario.

### Chemical transport model and aerosol microphysics model

We use the TOMCAT chemical transport model^49^ and the GLObal Model of Aerosol Processes (GLOMAP)^50^ to simulate the abundances of gas-phase and aerosol species. Both models operate at a horizontal resolution of 2.8×2.8°, with 31 pressure levels from the surface to 10 hPa. European Centre for Medium-Range Weather Forecasts (ECMWF; ERA-Interim) reanalyses data drives the meteorology in both models at 6-hourly intervals. Cloud fields for the year 2000 are taken from the International Satellite Cloud Climatology Project (ISCCP) archive^51^. With both models, simulations are performed for the year 2000 (with 1 year spin-up) and emissions of BVOCs are taken from the CLM.

We use GLOMAP-mode to simulate changes to the size and number of climatically relevant particles in the atmosphere when the source of biogenic SOA is altered^20^. GLOMAP-mode is a two-moment model, carrying particle number and composition details in five log-normal size modes: hydrophilic nucleation, Aitken, accumulation and coarse modes, and a non-hydrophilic Aitken mode. GLOMAP includes representations of particle formation and growth (via coagulation, condensation and cloud processing), as well as removal via wet and dry deposition. Particle phase material is classified as either sea-salt, sulphate, black carbon (BC) or particulate organic matter (POM; containing both primary and secondary organic species). Anthropogenic emissions (BC, POM and sulphur dioxide; SO_2_) for the year 2000 from fossil and biofuel combustion are taken from refs.^52,53^. Biomass burning emissions (BC, POM and SO_2_) are from the Global Fire Emissions Database (GFEDv3; ref.^54)^ inventory for the year 2000. Marine emissions of dimethyl-sulphide (DMS) are calculated using DMS sea surface concentrations^55^ along with a sea-to-air transfer velocity^56^. SO_2_ emissions from both continuous^57^ and explosive^58^ volcanic eruptions are included.

Gas-phase BVOCs (monoterpenes and isoprene) are oxidised by O_3_, OH and NO_3_ to generate secondary organic products. These reactions proceed with rate constants and molar yields (13% for monoterpenes and 3% for isoprene) from ref.^20^. The secondary organic products generated are assumed to be non-volatile and condense irreversibly onto existing particles in proportion to their Fuchs–Sutugin-corrected surface area^59^.

The new particle formation rate at 1.5 nm (J*) is parameterised according to ref.^15^ (Eq. 1) with *k* = 5 × 10^−13^ s^−1^; here NucOrg is the nucleating organic species. Subsequent growth of newly formed particles up to 3 nm is parameterised according to ref.^60^.

1 $$J_{{\mathrm{ORG}}}^\ast = k\left[ {{\mathrm{H}}_{\mathrm{2}}{\mathrm{SO}}_4} \right]\,\left[ {{\mathrm{NucOrg}}} \right]$$

In the standard configuration of GLOMAP-mode^50^, one gas-phase tracer is used to represent the oxidation products of monoterpenes and isoprene^20^. For this work, an additional gas-phase tracer is added to track the products of monoterpene and isoprene oxidation independently. The product of monoterpene oxidation contributes to both new particle formation (as NucOrg) and condensational growth, while the product of isoprene oxidation contributes only to condensational growth.

GLOMAP takes offline oxidants from equivalent deforestation simulations performed with the TOMCAT chemical transport model. This ensures that the gas-phase oxidant concentrations are consistent with the deforestation scenario. Monthly-mean oxidant concentrations (O_3_, OH, NO_3_, HO_2_ and H_2_O_2_) are read in at 6-h intervals; this simplification means that simulated changes to aerosol processes, due to deforestation, will not alter tropospheric chemistry. O_3_, OH and NO_3_ take part in the oxidation of BVOCs and formation of secondary organic aerosol. HO_2_ and H_2_O_2_ control the in-cloud oxidation of SO_2_, as described in ref.^50^; H_2_O_2_ is treated semi-prognostically and is replenished by HO_2_ self-reaction.

To simulate the impact of deforestation on gas-phase tropospheric chemistry, we use the TOMCAT chemical transport model^61^. TOMCAT includes the ExTC extended VOC degradation chemistry, simulating the oxidation of several C_2_ to C_7_ hydrocarbons. Monoterpene oxidation is based on the MOZART-3 scheme^62,63^ while the Mainz Isoprene Mechanism^64^ is applied to isoprene oxidation. Gas-phase emissions for the year 2000 are taken from the IPCC Fifth Assessment Report (AR5) anthropogenic inventory^65^ and the GFEDv3.1 inventory^54^. In addition to the BVOCs emitted by vegetation (calculated offline using the CLM), soil and marine emissions are included from the POET inventory^66^. A diurnal cycle is imposed on isoprene emissions within the model to reflect their dependence on daylight. Lightning emissions of NO_x_ are coupled to convection and calculated online. Methane (CH_4_) emission sources include EDGARv3.2 anthropogenic^67^, wetland and rice^68^, GFEDv3.1 fire^54^, and other natural emissions (treated as in ref.^69^). CH_4_ is emitted into the boundary layer of the model and at each time step surface concentrations are scaled to match a global mean concentration of 1800 ppbv; this approach generates a realistic spatial distribution, consistent with high and low emission regions. An offline aerosol size distribution from the GLOMAP model^50^ is used to calculate loss of N_2_O_5_ by aerosol uptake; this does not vary between our different deforestation scenarios.

Dry deposition of both gas- and particle-phase species is affected by characteristics of the land-surface. To simulate deforestation in GLOMAP, the characteristic radius and roughness length applied to the deforested land area were adjusted from forested values (0.1–2 m and 5 mm respectively) to appropriate values for grass (0.1 m and 2 mm respectively), following ref.^70^. In TOMCAT, the land type classification map is altered under the deforestation scenarios by converting the forest cover type to the crop/shrub/grass cover type, giving a less efficient dry deposition velocity for species including O_3_.

We use an 11-year GLOMAP simulation for the period 1997–2007 to provide information on the interannual variability in aerosol radiative effects. This simulation includes variability in both natural and anthropogenic aerosol emissions.

### Comparison to observations

In the boreal region, we compare GLOMAP output to monthly mean size distribution data collected (from 1996 to 2011) using a differential mobility particle sizer^71^, and composition data collected using an aerosol chemical speciation monitor^72^ (from 2012 to 2014) at Hyytiälä, Finland (61.9°N, 24.3°E)^73^. Particle size distributions are used to calculate the number concentration of particles with dry diameter greater than 80 nm (N_80_).

In the tropics, we compare GLOMAP to measurements made at the TT34 tower, 60 km north-northwest of Manaus (2.60°S, 60.21°W). Monthly mean size distribution data collected during 2008–2009 using a scanning mobility particle sizer (SMPS)^74,75^ was used to derive N_80_ concentrations; we include only SMPS data that could be validated (and agreed to within 15%) by comparison to particle number concentrations measured with a condensation particle counter. We also compare GLOMAP to composition data collected at the same site during 2008 as part of the European Integrated Project on Aerosol Cloud Climate Interactions (EUCAARI)^76^. At Manaus, we restrict our comparisons to the wet season (Jan-Jun) since particle mass and number concentrations in the dry season are dominated by transported regional biomass burning aerosol^75^.

### Calculation of radiative effects

We use the Suite Of Community RAdiative Transfer codes based on Edwards and Slingo^77^ (SOCRATES) to evaluate the radiative impact of deforestation induced changes to atmospheric composition and the land-surface. We operate SOCRATES with nine bands in the longwave (LW) and six bands in the shortwave (SW). We take monthly mean temperature and water vapour concentrations from ECMWF re-analysis data and use cloud fields for the year 2000 from the ISCCP-D2 archive^51^. Taking this approach, we have previously demonstrated that the sensitivity of direct and indirect aerosol radiative effects to using either a single year or a multi-annual mean cloud climatology is small^19^.

### Aerosol radiative effects

We use SOCRATES to calculate aerosol radiative effects by evaluating the difference in net (SW + LW) top-of-atmosphere all-sky radiative flux between each of our deforestation experiments and the control simulation. We compute the aerosol optical properties (scattering and absorption coefficients and the asymmetry parameter) for each size mode and spectral band in order to determine the direct radiative forcing (DRF) for each deforestation experiment^78^. The first indirect radiative forcing (IRF), or cloud albedo effect, is determined from the change to cloud droplet number concentration (CDNC) associated with each deforestation experiment. This approach has been described in previous studies^19,20^.

We use the monthly mean aerosol size distribution and the hygroscopicity parameter (*κ*) approach^79^ to calculate cloud droplet number concentrations^80^, assuming a uniform updraught velocity of 0.3 m s^−1^ over land and 0.15 m s^−1^ over sea. We assign the following *κ* values to our simulated aerosol components: sulphate (0.61, assuming ammonium sulphate), black carbon (0.0), sea-salt (1.28), and particulate organic matter (0.1). While there is considerable uncertainty associated with the hygroscopicity of organic material observed in the atmosphere, *κ* values close to 0.1 have frequently been reported for secondary organic aerosol produced from BVOC oxidation^81,82^. A multi-component *κ* is obtained by volume weighting the *κ* values of each component.

We assume a uniform control cloud droplet effective radius (*r*_e1_) of 10 µm to maintain consistency with the ISCCP derivation of liquid water path. For each deforestation experiment an effective radius (*r*_e2_) is calculated using monthly mean cloud droplet number fields CDNC_1_ and CDNC_2_ (as in Eq. 2, where CDNC_1_ represents the control simulation, and CDNC_2_ represents the deforested scenario).

2 $$r_{{\mathrm{e}}2} = r_{{\mathrm{e}}1} \times \left[ {\frac{{{\mathrm{CDNC}}_1}}{{{\mathrm{CDNC}}_2}}} \right]^{\frac{1}{3}}$$

The first IRF due to deforestation is then calculated by comparing the net top-of-atmosphere all-sky radiative fluxes obtained using the varying *r*_e2_ values, to those of the control simulation with fixed *r*_e1_. We do not calculate the second aerosol indirect, or cloud lifetime, effect in these offline experiments.

### O_3_ and CH_4_ radiative effects

To calculate the radiative forcing associated with deforestation-induced changes to tropospheric O_3_ concentrations, we use the radiative kernel approach developed by ref.^30^. O_3_ radiative effects calculated using the kernel approach agree well with those calculated using the SOCRATES radiative transfer model, both when O_3_ concentrations retrieved from Tropospheric Emission Spectrometer (TES) satellite measurements are used and when O_3_ concentrations are taken from the TOMCAT model^30^. Deforestation-induced changes to O_3_ concentration will alter the production of the hydroxyl radical (OH), and therefore CH_4_ concentrations. In turn, this will affect peroxy radical production and feedback on to O_3_ concentrations. The change in O_3_ concentration associated with this primary mode of tropospheric photochemistry is calculated following ref.^83^; we then apply a value of 0.032 W m^−2^ DU^−1^ (following ref.^24^) to diagnose a RF. We add this primary mode response to the RF calculated directly from O_3_ changes using the radiative kernel.

Our 1-year TOMCAT simulations do not allow for a direct calculation of the impact of a changing source of BVOCs on CH_4_ concentrations, owing its long atmospheric lifetime (approximately 10 years). As such, we use the concentration of OH as a proxy to infer changes to CH_4_ lifetime, and therefore concentration. The change in global annual tropospheric mean (CH_4_ reaction weighted, using a climatological tropopause)^84^ OH concentration is used to estimate the change to tropospheric chemical CH_4_ lifetime, and hence steady-state CH_4_ concentration^85,86^, assuming a feedback factor of 1.4 (ref.^87)^. The change in steady-state CH_4_ concentration is then used to quantify the global annual mean radiative forcing^88^ associated with each deforestation scenario, assuming a present-day N_2_O concentration of 324.2 ppb (ref.^35)^.

### Calculation of CO_2_ and surface albedo radiative effects

To understand the importance of the radiative forcings we calculate from changes in SLCFs, we also estimate the radiative impacts of altered atmospheric CO_2_ and surface albedo associated with our deforestation scenarios.

Changes to atmospheric CO_2_ concentration, for 100 years after deforestation were taken from ref.^9^, in which the Lawrence Livermore National Laboratory INCCA (Integrated Climate and Carbon) model was used to assess various impacts of simulated global and regional (using the same latitude bands as this study) forest removal in the year 2000. When forests are replaced by grass in these simulations, the carbon they stored is gradually added to the litter pool. The simulations proceed for 100 years following deforestation, allowing the carbon released (a total of 818 Pg(C) over 100 years) to partition amongst the atmosphere, ocean and land carbon sinks. Anthropogenic CO_2_ emissions follow the SRES A2 scenario^89^ from 2000 to 2100. The radiative forcing due to a change in CO_2_ concentration is calculated as in Eq. 2 following ref.^88^, where *C*_def_ represents the atmospheric CO_2_ concentration (in ppmv) in the deforestation scenario, and *C*_con_ that in the control scenario.

3 $${\mathrm{RF}}_{{\mathrm{CO}}_2} = 5.35\ln \frac{{C_{{\mathrm{def}}}}}{{C_{{\mathrm{con}}}}}$$

The ratio of SW radiation incident upon and reflected by the surface, for each grid cell in the CLM, was used to determine the SW surface albedo for each deforestation scenario. In the CLM, LAIs are adjusted for burial by snow, according to snow depth and vegetation height, which in turn affects the amount of SW radiation reflected by the surface. The radiative transfer model was then used to evaluate the radiative impact of the change in albedo between the various scenarios by comparing the net top-of-atmosphere flux.

We note that taking CO_2_ concentrations from ref.^9^ means that the simulated changes to the land surface may have been slightly different (in terms of plant functional type distribution, assumed leaf area indices and stored biomass) to those assumed in the CLM experiments used to determine the RFs due to SLCFs and albedo. Additionally, since present-day gas-phase and aerosol emissions, and concentrations are assumed when calculating the RFs associated with changes to SLCFs, we note that these values would alter if calculated assuming emissions and concentrations representative of a future period.

### Calculation of global temperature potentials

To estimate the surface temperature change, the RF scenarios are incorporated into a simple two-layer energy balance model used in the Global Temperature Potential (GTP) derivation^90^, choosing an equilibrium climate sensitivity of 3 K; the mid-range value from the latest IPCC assessment report. As climate sensitivity is uncertain, the temperature changes computed here are illustrative.

### Data availability

The data sets generated, and analysed, during the current study are available from the corresponding author upon request.

## Electronic supplementary material

Supplementary Information
