# Supplementary material for: Impact on short-lived climate forcers increases projected warming due to deforestation
Source: Nat Commun. 2018 Jan 11;9:157. doi: 10.1038/s41467-017-02412-4 (PMC5764971; doi:10.1038/s41467-017-02412-4)
Supplement: Supplementary file 1 — Supplementary Information [file 41467_2017_2412_MOESM1_ESM.pdf]

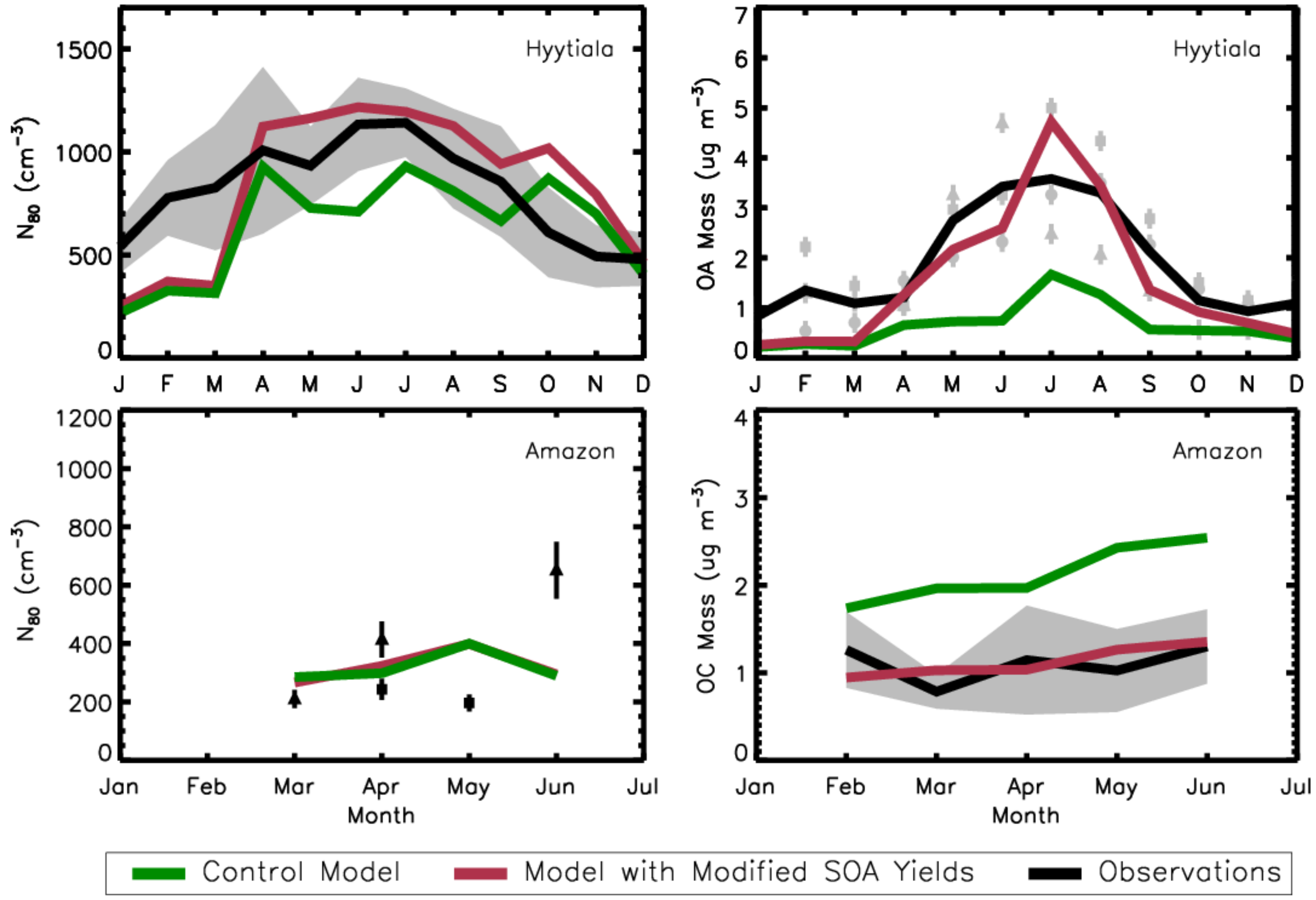

Supplementary Figure 1: Comparison of aerosol simulated by GLOMAP to observations taken from a boreal forest location at Hyytiälä in Finland (top panels) and a tropical forest location in the Brazilian Amazon (bottom panels). Comparison shows N_{80} concentrations (left panels) and organic aerosol (OA) and organic carbon (OC) mass (right panels). At Hyytiälä we show observed multi-annual (1996-2011) monthly mean N_{80} (solid black line) and standard deviation in the observed multi-annual monthly mean (grey shading) and observed multi-annual (2012-2014) monthly mean OA mass (solid black line) with monthly mean for each individual year (grey symbols; ± 0.2 µg m^{-3} standard error shown with error bars). In the Amazon we show observed (2008-2009) monthly mean N_{80} (symbols; ±15% uncertainty shown as error bars) and observed monthly mean OC mass (solid black line) and standard deviation in the observed monthly mean (grey shading), during the wet season only. Simulated values are shown for the control model (green line) and with modified SOA yields (red line); see Methods for further details.

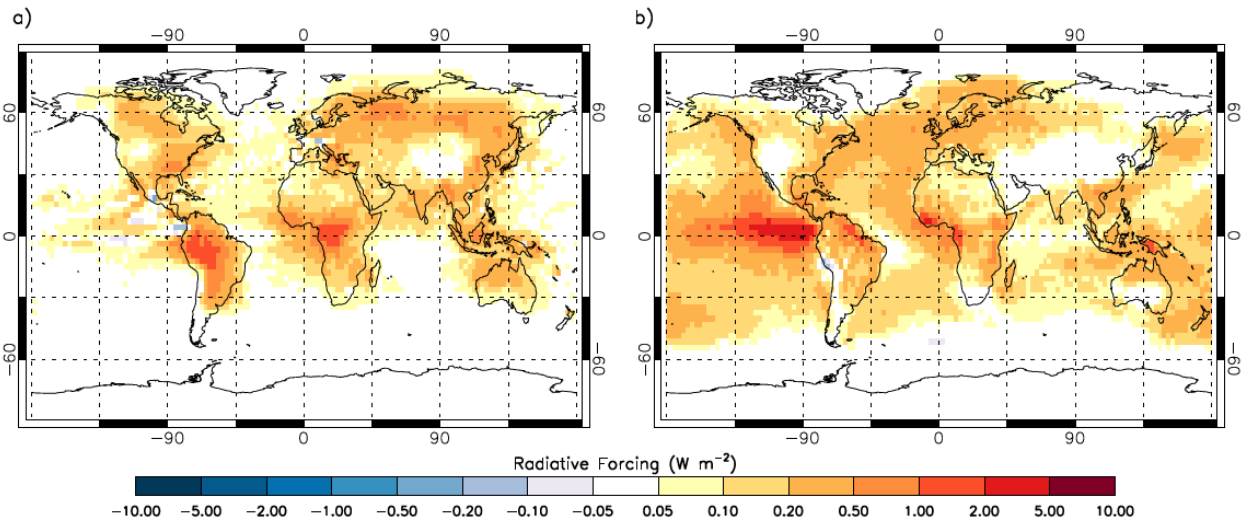

Supplementary Figure 2: Global annual mean direct radiative forcing (a) and first indirect radiative forcing (b) using modified SOA yields.

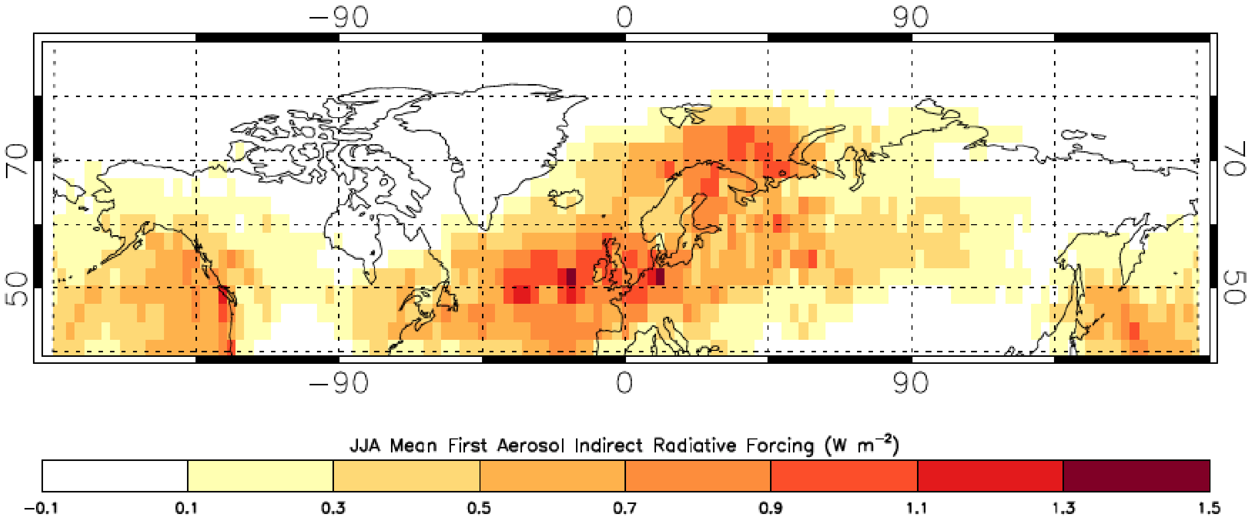

Supplementary Figure 3: Northern hemisphere summertime (June-July-August) mean first indirect RF due to global deforestation, using modified SOA yields.

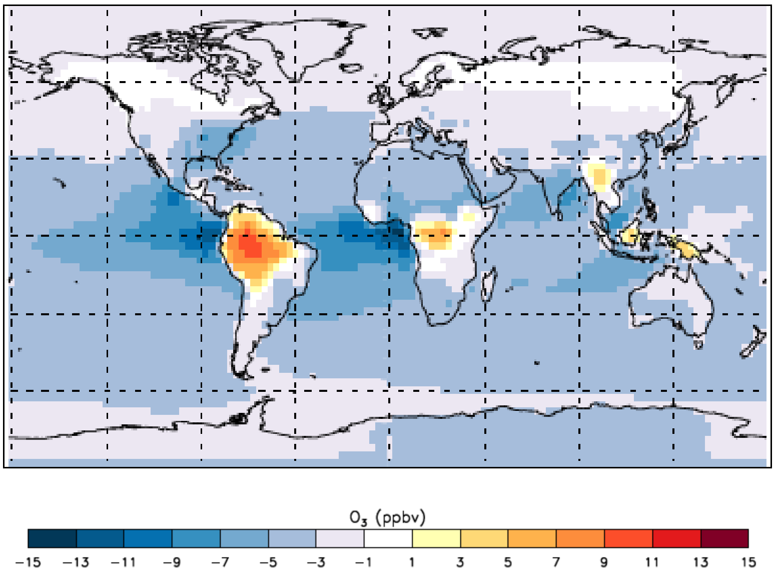

Supplementary Figure 4: Annual mean change to O_3 concentration (ppbv) in the model surface layer, calculated using the TOMCAT model, due to global deforestation.

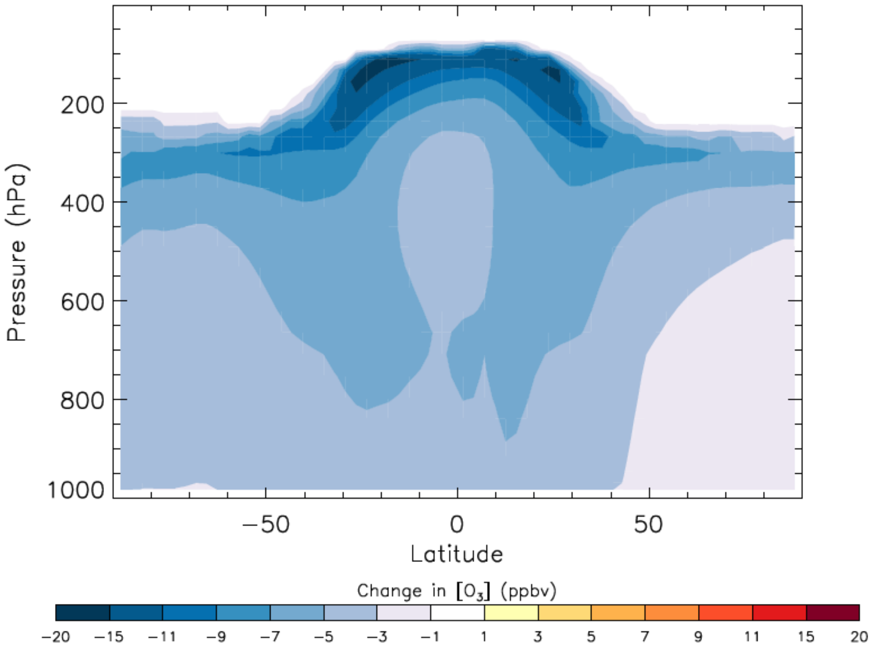

Supplementary Figure 5: Annual zonal mean change to O_3 concentration (ppbv), calculated using the TOMCAT model, due to global deforestation

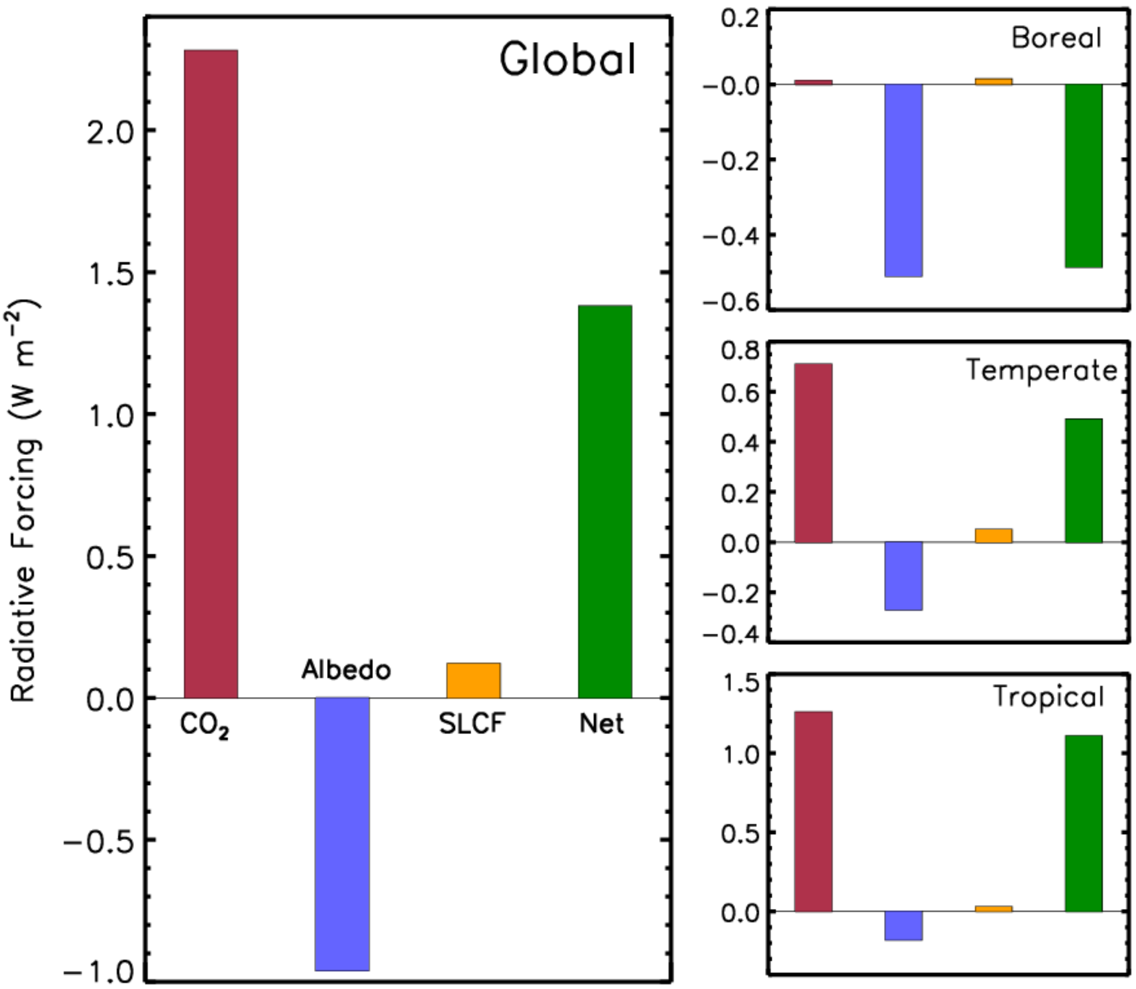

Supplementary Figure 6: Global annual mean radiative forcings (RF) due to changes in the concentrations of CO_2 (red), changes to surface albedo (blue), and changes to the concentrations of SLCFs (orange), under global (left) and regional (right) deforestation scenarios.

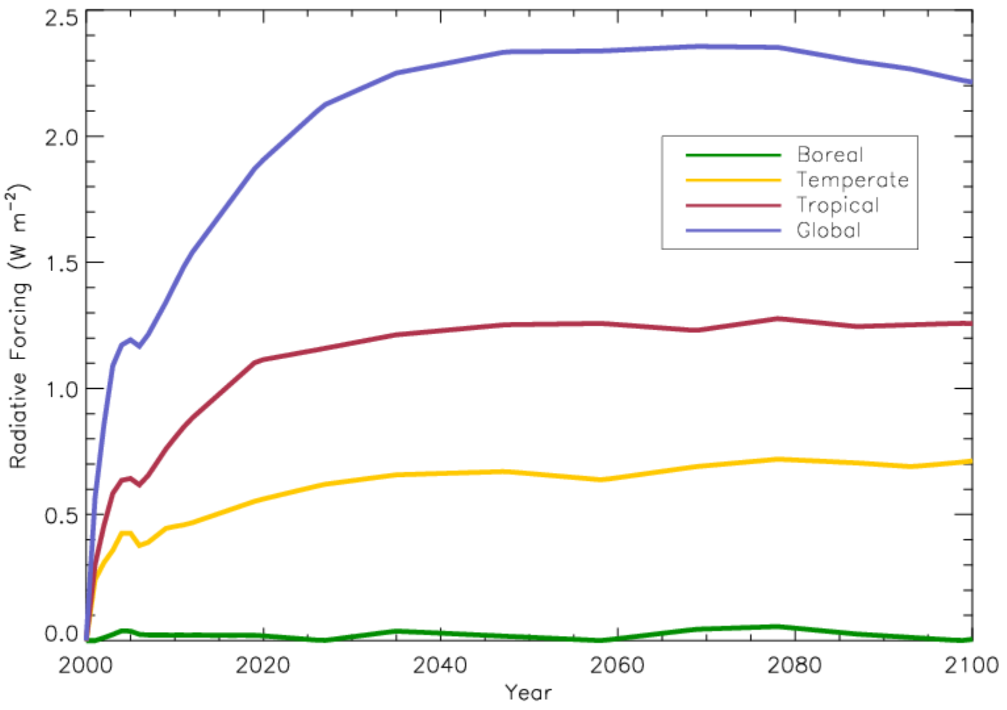

Supplementary Figure 7: Radiative forcing from CO_2, for 100 years following deforestation, using CO_2 concentration scenarios presented in Figure 1 of ref[1].

Supplementary Tables:

Supplementary Table 1: BVOC emission and SOA production totals for each simulation; for each deforestation scenario the change in BVOC emission and SOA production relative to the control simulation is given.

	Global annual total					
	**Isoprene emission** ($Tg(C)\ a^{-1}$) **and % change from control**		**Total monoterpene emission** ($Tg(C)\ a^{-1}$) **and % change from control**		**SOA generated** ($Tg(SOA)\ a^{-1}$) **and % change from control**	
Control *(value for simulation with modified SOA yields given in brackets)*	480		140		40 (31)	
Boreal deforestation	475	-1%	133	-5%	38	-3%
Temperate deforestation	412	-14%	119	-15%	34	-15%
Tropical deforestation	133	-72%	36	-74%	11	-73%
Global deforestation *(value for simulation with modified SOA yields given in brackets)*	60	-87%	8	-94%	3 (4)	-91% (-86%)
RCP8.5 in 2100	462	-4%	135	-4%	38 (29)	-4% (-3%)

Supplementary Table 2: Global annual mean radiative forcings ($W\ m^{-2}$) due to changes in the concentration of SLCFs; values for aerosol radiative forcings obtained from the modified SOA yield experiments are given in brackets.

	**Direct RF** ($W\ m^{-2}$)	**First Indirect RF** ($W\ m^{-2}$)	**O_3 RF** ($W\ m^{-2}$)	**CH_4 RF** ($W\ m^{-2}$)	**Combined SLCF RF** ($W\ m^{-2}$)
Boreal deforestation	0.00 (0.02)	0.02 (0.01)	0.00	0.00	0.01 (0.03)
Temperate deforestation	0.03 (0.03)	0.06 (0.06)	-0.03	-0.01	0.05 (0.05)
Tropical deforestation	0.13 (0.07)	0.10 (0.09)	-0.14	-0.06	0.03 (-0.04)
Global deforestation	0.17 (0.12)	0.20 (0.19)	-0.17	-0.07	0.12 (0.07)
RCP8.5 in 2100 *(given to 3 d.p.)*	0.006 (0.004)	-0.001 (-0.001)	-0.002	-0.003	0.000 (-0.003)

Supplementary Discussion:

For our baseline simulation we require an aerosol state that is consistent with observations in order to quantify an appropriate response to deforestation. Supplementary Figure 1 compares simulated and observed aerosol concentrations at boreal and tropical forest locations where the aerosol size distribution, and its seasonal variability, are strongly influenced by the emission of BVOCs. The number of aerosols with dry diameter greater than 80 nm (N_{80}) may be used as a proxy for cloud condensation nuclei (CCN) concentration and the impacts of BVOCs on clouds, whilst fine particulate mass may be used as a proxy for the direct aerosol effect.

In the boreal region, we compare GLOMAP output with measurement data collected at Hyytiälä, Finland (see Methods). At Hyytiälä, a seasonal cycle in N_{80} is observed, with summertime (JJA) N_{80} concentrations a factor of two higher than wintertime (DJF); when biogenic SOA is not included, GLOMAP is unable to capture this seasonal variation[2]. In our control simulation, GLOMAP underpredicts summertime concentrations of N_{80} and organic mass (green lines in Supplementary Figure 1 *a* & *b*). To address these underpredictions, we perform a sensitivity study in which we increase the yield of SOA formation by a factor of 5 across boreal latitudes (red lines in Supplementary Figure 1 *a* & *b*); this leads to an increase in both N_{80} and organic mass throughout much of the year, and better agreement with the measurements. In performing these sensitivity studies we appreciate that there are multiple factors that can cause uncertainty in aerosol properties[3] and more specifically within the formation process of secondary organic aerosol[4, 5, 6, 7]. However, consistent underprediction in both N_{80} and organic mass concentrations strongly suggests that the control simulation does not include sufficient secondary organic aerosol at this site.

In the tropics, we compare GLOMAP output to measurement data collected near Manaus, Brazil (see Methods). Over the Amazon, the model captures the N_{80} concentrations during the wet season well (Supplementary Figure 1*c*), but overpredicts the observed organic mass concentration by around a factor of 2. To address this overprediction, we decrease the yield of SOA formation by a factor of 2 (red lines in Supplementary Figure 1 *c* & *d*) across tropical latitudes and see better agreement between the modelled and observed organic mass. Decreasing the SOA yield in the tropics has little impact on simulated N_{80} in the Amazon, due to non-linear relationships between SOA formation and N_{80} (ref[2]), and model performance is similar to the control simulation.

The global annual mean tropospheric burden of O_3, simulated by TOMCAT for our control scenario is 336 Tg O_3, within the range simulated by the ACCMIP models (337 ± 23 Tg O_3; ref[8]). The simulated O_3 column burden shows good agreement with measurements made by the Tropospheric Emission Spectrometer (TES)[9] and O_3 concentrations in the upper troposphere lie within the observed range of ozonesonde data in the

tropics[10]. However, the model overestimates O_3 in the mid to high latitudes by around 10-20 ppbv, most likely due to too great an influence from stratospheric ozone in these regions[10].

The global annual tropospheric mean (CH_4 reaction weighted[11]) OH concentration simulated by TOMCAT is 1.36×10^6 molecules cm^{-3}. This gives a tropospheric chemical CH_4 lifetime (calculated according to ref[12]) of 7.6 years, which is towards the lower end of, but within, the range of values simulated by the ACCMIP models (7.1 – 13.9 years)[12].

Despite substantial summertime BVOC emissions at northern high latitudes, boreal deforestation has a small impact on the global annual BVOC emissions, reducing global isoprene emissions by 1% and global monoterpene emissions by 5%. Temperate deforestation reduces the global isoprene and monoterpene emissions by 14 and 15% respectively.

We also take account of changes in BVOCs other than isoprene and monoterpenes that influence gas-phase chemistry. However, we can assume that changes in OH and O_3 are mostly driven by isoprene and monoterpene emission reductions as the fractional changes in emissions are much larger. In our global deforestation scenario, other hydrocarbons (C_2H_4, C_2H_6, C_3H_6 and C_3H_8) are reduced by 17%, oxygenated hydrocarbons (CH_3COCH_3, CH_3OH and HCHO) are reduced by 39% and CO is reduced by 8%. This lower sensitivity to deforestation arises because the emission rates of the additional BVOCs in MEGAN are not as highly influenced by changes in the PFT.

Supplementary References:

1. Bala G, *et al.* Combined climate and carbon-cycle effects of large-scale deforestation. *PNAS* **104**, 6550-6555 (2007).

2. Scott CE, *et al.* The direct and indirect radiative effects of biogenic secondary organic aerosol. *Atmos Chem Phys* **14**, 447-470 (2014).

3. Lee LA, *et al.* The magnitude and causes of uncertainty in global model simulations of cloud condensation nuclei. *Atmos Chem Phys* **13**, 8879-8914 (2013).

4. Spracklen DV, *et al.* Aerosol mass spectrometer constraint on the global secondary organic aerosol budget. *Atmos Chem Phys* **11**, 12109-12136 (2011).

5. Weber RJ, *et al.* A study of secondary organic aerosol formation in the anthropogenic-influenced southeastern United States. *J Geophys Res* **112**, D13302 (2007).

6. Hoyle CR, *et al.* A review of the anthropogenic influence on biogenic secondary organic aerosol. *Atmos Chem Phys* **11**, 321-343 (2011).

7. Hallquist M, *et al.* The formation, properties and impact of secondary organic aerosol: current and emerging issues. *Atmos Chem Phys* **9**, 5155-5236 (2009).

8. Young PJ, *et al.* Pre-industrial to end 21st century projections of tropospheric ozone from the Atmospheric Chemistry and Climate Model Intercomparison Project (ACCMIP). *Atmos Chem Phys* **13**, 2063-2090 (2013).

9. Rap A, Richards NAD, Forster PM, Monks SA, Arnold SR, Chipperfield MP. Satellite constraint on the tropospheric ozone radiative effect. *Geophysical Research Letters* **42**, 5074-5081 (2015).

10. Monks SA, *et al.* The TOMCAT global chemical transport model v1.6: description of chemical mechanism and model evaluation. *Geosci Model Dev* **10**, 3025-3057 (2017).

11. Lawrence MG, Jöckel P, von Kuhlmann R. What does the global mean OH concentration tell us? *Atmos Chem Phys* **1**, 37-49 (2001).

12. Voulgarakis A, *et al.* Analysis of present day and future OH and methane lifetime in the ACCMIP simulations. *Atmos Chem Phys* **13**, 2563-2587 (2013).
